# The complete mitochondrial genome of the edible Basidiomycete mushroom *Phlebopus Portentosus*

**DOI:** 10.1080/23802359.2017.1383195

**Published:** 2017-10-05

**Authors:** Lili Jiang, Dan Yang, Yang Cao, Pengfei Wang, Yunrun Zhang, Ke-Qin Zhang, Jianping Xu, Ying Zhang

**Affiliations:** a State Key Laboratory for Conservation and Utilization of Bio-Resources in Yunnan, and Key Laboratory for Southwest Microbial Diversity of the Ministry of Education, Yunnan University, Kunming, Yunnan, P. R. China;; b Yunnan Institute for Tropical Crop Research, Jinghong, Yunnan, P. R. China;; c Department of Key Laboratory, The 2nd Affiliated Hospital of Kunming Medical University, Kunming, Yunnan, P. R. China;; d Department of Biology, Mc Master University, Hamilton, Ontario, Canada

**Keywords:** *Phlebopus portentosus*, mitogenome, phylogenetic relationship

## Abstract

The complete mitochondrial genome of *Phlebopus portentosus* was determined using Illumina sequencing. The circular genome is 42,963 bp in length with GC content of 21.37%. It contains 14 putative protein-coding genes, the ribosomal RNA subunits and 23 tRNAs, all located on the same strand. The evolutionary relationships between *P. portentosus* and other 23 representative basidiomycete species were revealed based on sequences at the 14 concatenated mitochondrial protein-coding genes.

*Phlebopus portentosus* is an artificially cultivatable boletus mushroom and the mitochondrion is an important essential organelle in mushrooms (Burger et al. 2003; Ji et al. 2011). Elucidating the structure and function of *P. portentosus* mitogenome is important for understanding its genetics, diversity and evolution. The genome sequence of *P. portentosus* has been reported (Cao et al. 2015). However, little is known about its mitogenome. Here, we report the mitogenome of *P. portentosus* and investigate the phylogenetic relationships of *P. portentosus* and other related species based on mitochondrial protein-coding genes.

The mitogenome sequence of *P. portentosus* was obtained from strain PP33 from the Institute of Tropical Crops of Yunnan Province (collected from Dongfeng Farmland which is 34 km south to Jinghong City, Xishuangbanna Autonomous Prefecture, Yunnan Province, China. N 21°42’ E 100°45. And the single spore isolate of *P. portentosus* was deposited as living cultures in China General Microbiological Culture Collection Center under the accession number: CGMCC 6240). The published mitogenome of *Ganoderma lucidumd* (GenBank ID: KP410262) was used as a reference. The mitogenome sequence of *P. portentosus* was identified by BLAST as scaffold54 (GenBank ID: KN880405) in the whole genome assembly and annotated using MFannot (http://megasun.bch.umontreal.ca/cgi-bin/mfannot/mfannotInterface.pl) and GLIMMER (https://www.ncbi.nlm.nih.gov/genomes/MICROBES/glimmer_3.cgi). tRNAs were annotated using tRNAscan-SE (Schattner et al. 2005). All ORFs were searched and identified by ORFFinder.

The mitogenome of strain PP33 is a typical circular DNA molecule of 42,963 bp in length with a GC content of 21.37%. Genome annotation identified ORFs that encode 14 conserved proteins, 23 tRNAs, 2 homing endonucleases, the small ribosomal RNAs (rns) and the large ribosomal RNA (rnl). The 14 conserved proteins include 7 subunits of NAD dehydrogenase (nad1-6 and nad4L genes), 3 cytochrome oxidases (cox1-3), apocyto chrome b (cob) and 3 subunits of ATP synthase (atp6, apt 8 and apt 9). The 23 tRNA genes covered all 20 standard amino acids. There were 5 introns distributed in this mitogenome, 4 of which were found in the cox1 gene: two were group I and the other two were group II. Phylogenetic analysis based on concatenated sequences of 14 conserved mitochondrial protein-coding genes of 23 Basidiomycetes was performed by Bayesian inference (BI). As shown in Figure 1. *P. portentosus* was a member of Agaricomycotina and closely related to Russulales and Polyporales. The inferred relationships using mitochondrial gene sequences were the same as those constructed based on sequences of nuclear genes (Garcia-Sandoval et al. 2011; Wu et al. 2014). The Genbank Accession number for Phlebopus Portentosus is MK571437.

**Figure 1.**
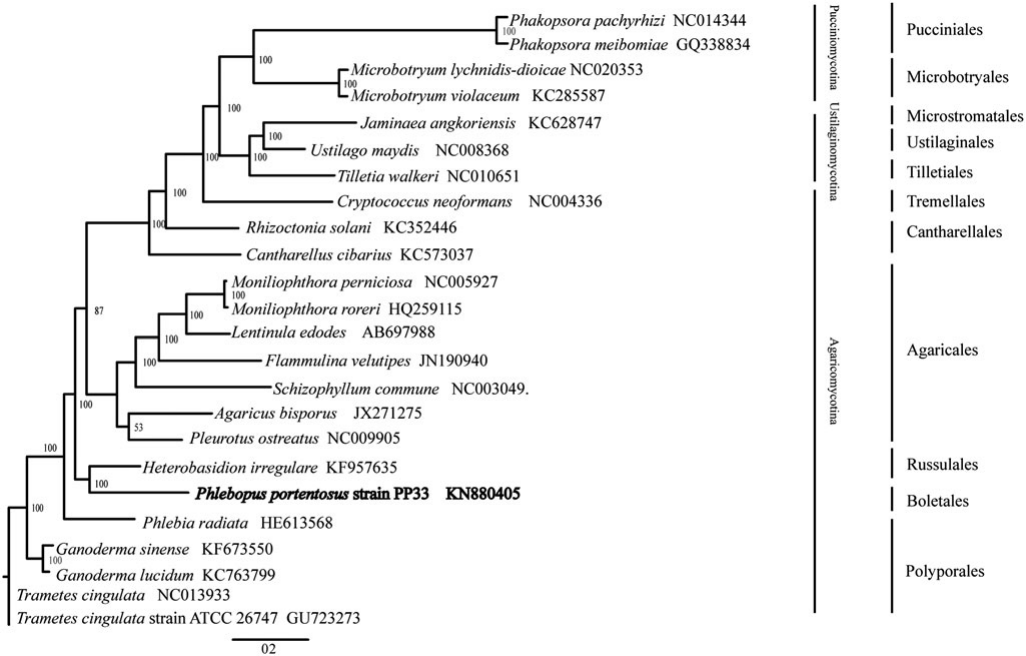
Phylogenetic relationships among 23 basidiomycete fungi inferred based on the concatenated amino acid sequences of 14 mitochondrial protein-coding genes. The 14 mitochondrial protein-coding genes were: nad1, nad2, nad3, nad4, nad4L, nad5, nad6, cox1, cox2, cox3, cob, atp6, atp8, atp9. The tree was generated using Bayesian inference (BI). Numerical values along branches represent statistical support based on 1000 randomizations.
